# Iron Supplementation in Suckling Piglets: How to Correct Iron Deficiency Anemia without Affecting Plasma Hepcidin Levels

**DOI:** 10.1371/journal.pone.0064022

**Published:** 2013-05-30

**Authors:** Rafał R. Starzyński, Coby M. M. Laarakkers, Harold Tjalsma, Dorine W. Swinkels, Marek Pieszka, Agnieszka Styś, Michał Mickiewicz, Paweł Lipiński

**Affiliations:** 1 Institute of Genetics and Animal Breeding, Jastrzębiec, Poland; 2 Department of Laboratory Medicine (LGEM 830), Radboud University Medical Center, Nijmegen, The Netherlands; 3 Hepcidinanalysis.com, Nijmegen, The Netherlands; 4 National Research Institute of Animal Production, Balice, Poland; 5 Mifarmex Ltd, Michałów-Grabina, Poland; Penn State Hershey Medical Center, United States of America

## Abstract

The aim of the study was to establish an optimized protocol of iron dextran administration to pig neonates, which better meets the iron demand for erythropoiesis. Here, we monitored development of red blood cell indices, plasma iron parameters during a 28-day period after birth (till the weaning), following intramuscular administration of different concentrations of iron dextran to suckling piglets. To better assess the iron status we developed a novel mass spectrometry assay to quantify pig plasma levels of the iron-regulatory peptide hormone hepcidin-25. This hormone is predominantly secreted by the liver and acts as a negative regulator of iron absorption and reutilization. The routinely used protocol with high amount of iron resulted in the recovery of piglets from iron deficiency but also in strongly elevated plasma hepcidin-25 levels. A similar protocol with reduced amounts of iron improved hematological status of piglets to the same level while plasma hepcidin-25 levels remained low. These data show that plasma hepcidin-25 levels can guide optimal dosing of iron treatment and pave the way for mixed supplementation of piglets starting with intramuscular injection of iron dextran followed by dietary supplementation, which could be efficient under condition of very low plasma hepcidin-25 level.

## Introduction

Iron deficiency is considered to be the most common mammalian nutritional deficiency [1] and is most prevalent in the neonatal period [2]. Neonatal IDA is particularly frequent and severe in pigs, regardless of the breed and the system of piglet rearing [3], [4]. Iron scarcity in piglets is the result of interplay of several distinct risk factors such as low level of iron stores, increased iron requirements, limited external supply and the immaturity of molecular mechanisms of iron absorption [5], [6]. For decades pigs have been selected for large litter size, high birth weight and fast growth, which resulted in greater body blood volume, red blood cells (RBC) count, and in consequence, in increased iron demands. Both hepatic iron reserves and the sow’s milk are therefore nowadays not sufficient to meet iron requirements in suckling piglets [3], [4], [7]. Moreover, the molecular machinery responsible for iron absorption in newborn piglets is not fully developed, and this may explain a reduced responsiveness of these animals to oral iron therapy [8], [9]. Consequently, the use of parenteral injection of exogenous iron to prevent deepening iron deficiency in suckling piglets has been well documented and is obligatory in pig breeding [10], [11], [12]. Various iron supplementation strategies (in terms of time and route of iron administration as well as the amount and the form of supplemental iron) have been tested and many of them proved to be beneficial in correcting IDA in newborn piglets as evaluated by measuring their RBC indices and serum iron status [10], [11], [12], [13], [14], [15].

Over the past eleven years major insights have been made into the role of hepcidin in the tuning of systemic iron homeostasis [16], [17]. Hepcidin is a peptide hormone, mainly secreted by the liver, which acts as a negative regulator of iron absorption and its reutilization by macrophages of the reticuloendothelial system (RES). By binding to ferroportin, the only known iron exporter, hepcidin causes ferroportin internalization and degradation, hence decreasing the absorption of dietary iron by enterocytes and the release of iron from macrophages that have engulfed senescent RBCs, into the plasma. Inappropriate low hepcidin levels cause the iron overload disorders, whereas increased hepcidin expression leads to anemia due to the insufficient intestinal iron absorption and iron arrest in the RES [16].

Considering that hepcidin is up-regulated by high iron concentrations in the plasma and the liver [16], we aimed in this study to establish an optimal strategy of iron supplementation in piglets, which could both improve RBC indices and minimize dysfunctional induction of hepcidin expression that could potentially have an adverse effect on iron utilization.

The intramuscular injection of large amount of iron dextran to suckling piglets is a relatively simple therapy, which in general, efficiently corrects iron deficiency anemia (IDA), widely occurring in pig neonates, and therefore is routine practice in pig breeding [10], [11], [12]. We have recently demonstrated that excessive loading of piglets with iron dextran induces *hepcidin* expression at the mRNA level in the liver, and may perturbate the utilization of iron released from this compound by blocking ferroportin [10], [20]. It has been well established that after injection, iron dextran is rapidly taken from plasma and deposited in the reticuloendothelial system [21]. The elemental iron is then released from the complex with polyisomaltose by macrophages (mainly by Kupffer cells) and successively re-enters plasma via a ferroportin-dependent pathway. Next, the blood flow iron is complexed with transferrin (Tf) and transported to the bone marrow to allow hemoglobin synthesis. It seems that the optimal iron supplementation in piglets should meet two main criteria: 1) the delivery of sufficient amount of iron for erythropoiesis; 2) the avoidance of a dysfunctional increase in plasma hepcidin levels.

We have recently reported that by both reducing the amount of supplemented iron and modifying the timing of its dosage, we can improve the hematological status of piglets while attenuating their hepatic *hepcidin* mRNA levels [10]. Here, we have developed a mass spectrometry assay to quantify bioactive hepcidin-25 in piglet plasma and assessed different protocols of piglet supplementation with iron dextran for their ability to stimulate recovery from neonatal IDA without inducing an unfavorable increase in plasma hepcidin.

## Materials and Methods

### Ethics Statement

Use of animals in the experiment and all procedures were approved by the Second Local Ethical Committee on Animal Testing at the Institute of Pharmacology in Krakow (permission no. 975).

### Animals, Experimental Design, and Blood Samples Collection

**Figure 1:**
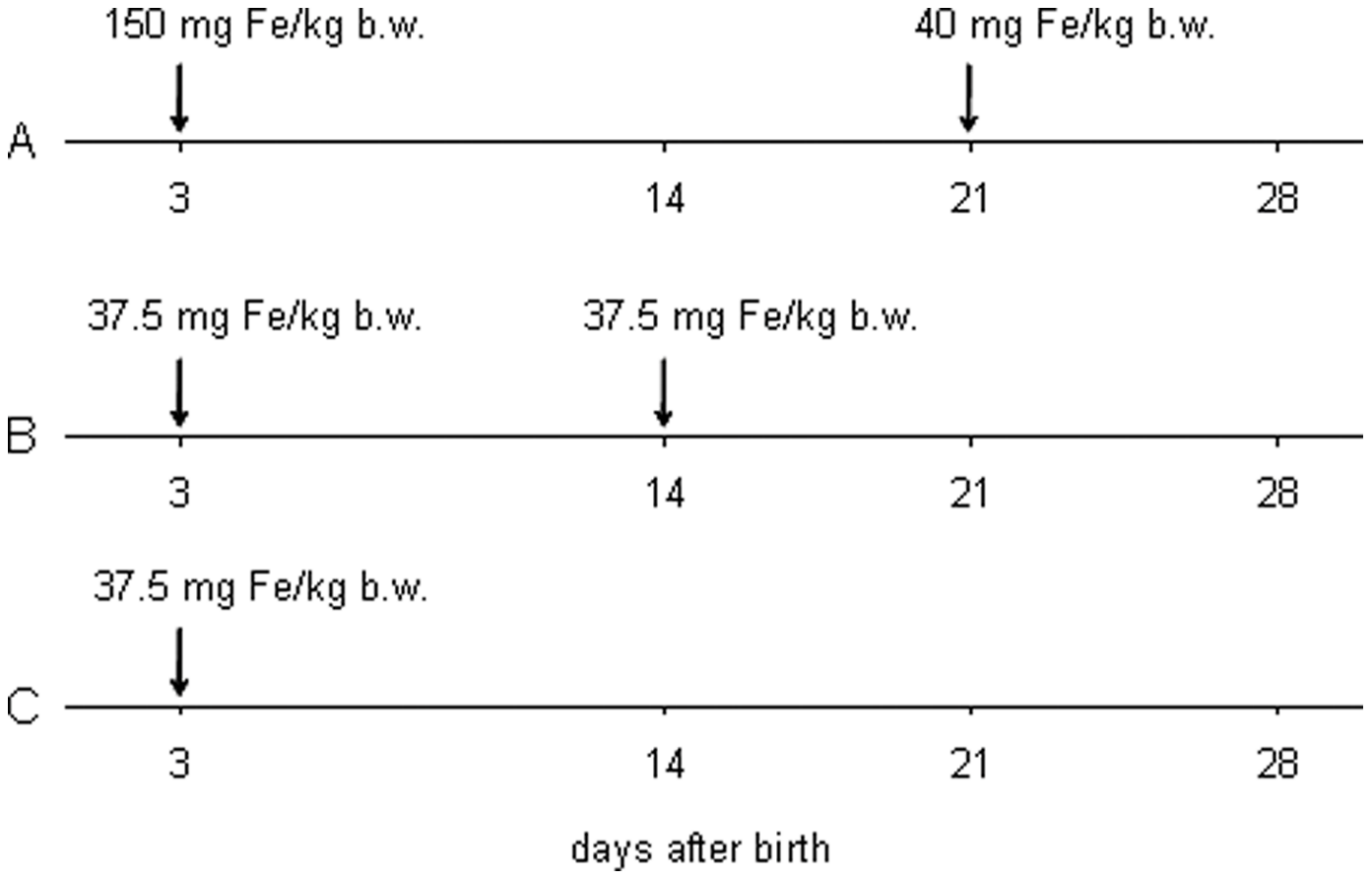
Experimental design scheme. 3 various protocols of iron dextran administration in piglets are indicated as A, B and C. Iron dextran was injected to piglets on days indicated by arrows. Blood samples were collected on days 3, 14, 21 and 28 after birth. When iron dextran injection and blood collection fell on the same day, blood was always drawn before iron administration.

Experiments were conducted at the pig farm Brzezie belonging to the National Research Institute of Animal Production (Balice, Poland). A total of 27 Polish Landrace × Polish Large White piglets housed in standard conditions (approx. 70% humidity and a temperature of 22±2°C in standard cages with straw bedding) were used in the experiments. During the 28-day experiment sows were allowed to nurse their piglets. The feed (Prestarter, was manufactured at the feed mill of the Experimental Station of the National Research Institute of Animal Production in Brzezie; iron content 0.75 mg Fe/kg feed mixture) was offered to piglets from day 7 to day 28. Piglets were taken from 3 litters delivered by 3 multipara sows. They were allotted to one of following experimental groups (9 piglets per group) on the basis of balanced body weight (b.w.) at birth (Figure 1): A) piglets routinely supplemented by intramuscular injection with 150 and 40 mg Fe/kg b.w. on days 3 and 21 postpartum, respectively; B) piglets supplemented with 37.5 mg Fe/kg b.w. on days 3 and 14 postpartum; C) piglets supplemented with 37.5 mg Fe/kg b.w. on day 3 postpartum only. Iron was administered to piglets by intramuscular injection in the neck in the form of iron dextran (FeDex), a complex of ferric ions with low molecular weight dextran (Suiferron, Mifarmex Ltd, Michałów-Grabina, Poland or Ferran 100, Vet-Agro, Lublin, Poland). Blood samples for analyses were taken from all piglets on days 3, 14, 21, and 28 of life. At the same periods all animals were weighed. Blood was drawn by venipuncture of the jugular vein (*Vena jugularis externa*) into tubes coated with heparin or EDTA as anticoagulants. Heparinized blood was immediately spun down (at 4°C, 2000 rpm, 10 min) to collect plasma. Plasma samples were aliquoted and stored at −80°C. EDTA-whole blood was used for immediate hematological analyses.

### Hematological Analysis

Red blood cells (RBC) parameters such as RBC count, hemoglobin level (HGB), hematocrit (HCT), mean cell volume (MCV), mean corpuscular hemoglobin (MCH), and mean corpuscular hemoglobin concentration (MCHC) were determined using an automated AVIDIA 2010 analyzer (Siemens, Germany).

### Plasma Iron Parameters

Plasma iron concentration and total iron binding capacity (TIBC) were determined by colorimetric measurement of the absorbance of the iron-chromazurol complex at 630 nm (Alpha Diagnostic, Poland). Percent of transferrin (Tf) saturation by iron was calculated according to the following formula: TSAT = [plasma iron/TIBC] × 100.

### Plasma Hepcidin-25 Quantification

**Figure 2:**
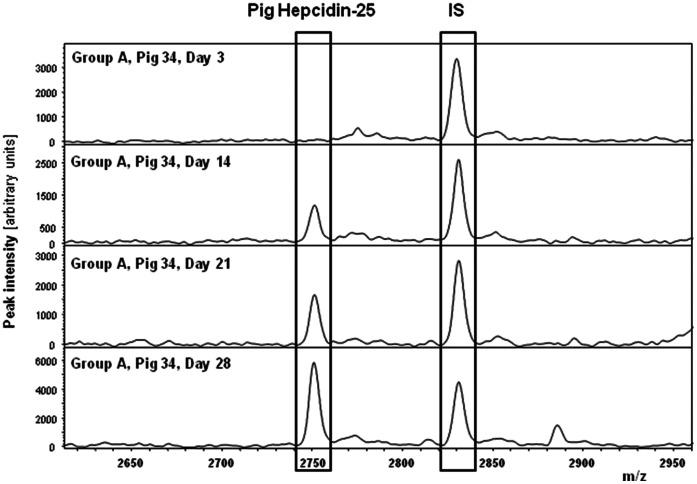
Pig Hepcidin-25 quantification by mass spectrometry. Hepcidin-25 measurements in piglet plasma were performed by peptide enrichment through weak cation exchange chromatography coupled to time-of-flight mass spectrometry (WCX-TOF MS). Note that quantification is based on the relative intensity of the piglet hepcidin peak with mass/charge ratio (m/z) of 2750 to that of the synthetic internal standard (IS) that is spiked in the know concentration of 10 nM to each sample prior to sample work-up. The four spectra illustrate the appearance of hepcidin-25 upon iron injection in group A, pig № 34 at days 3 (baseline), 14, 21 and 28.

Piglet plasma hepcidin-25 measurements were performed by a combination of weak cation exchange chromatography and time-of-flight mass spectrometry (WCX-TOF MS), as described previously for human plasma samples [17], [18], [19]. Peptide spectra were generated on a Microflex LT matrix-enhanced laser desorption/ionisation TOF MS platform (BrukerDaltonics). When piglet samples were applied, this procedure yielded a peak with mass/charge ratio (m/z) of 2750, which corresponds to the theoretical mass of pig hepcidin-25 (2749.4 Da), assuming that 4 intra-molecular disulphide bridges are present as is the case for all other known hepcidin molecules (Figure 2). The identity of this peak was further confirmed by its specific disappearance from the mass spectrum by pre-incubation of piglet plasma samples with anti-hepcidin molecules (data not shown). A synthetic human hepcidin-25 peptide (Peptide International Inc.) was used as internal standard for quantification. Piglet plasma hepcidin-25 concentrations were expressed as nmol/L (nM). The lower limit of detection of this method was 1 nM.

### Statistical Analysis

Data are presented as mean values ± SD. Statistical analysis of results was performed using one-way analysis of variance (one-way ANOVA). The significance of differences was verified by Tukey’s test using Statgraphics 5.1 program (Manugistics, USA). Statistically significant differences between parameters of piglets from different groups on a given day of experiment were denoted by different capital and small letters at P≤0.01 and P≤0.05, respectively.

## Results and Discussion

**Figure 3:**
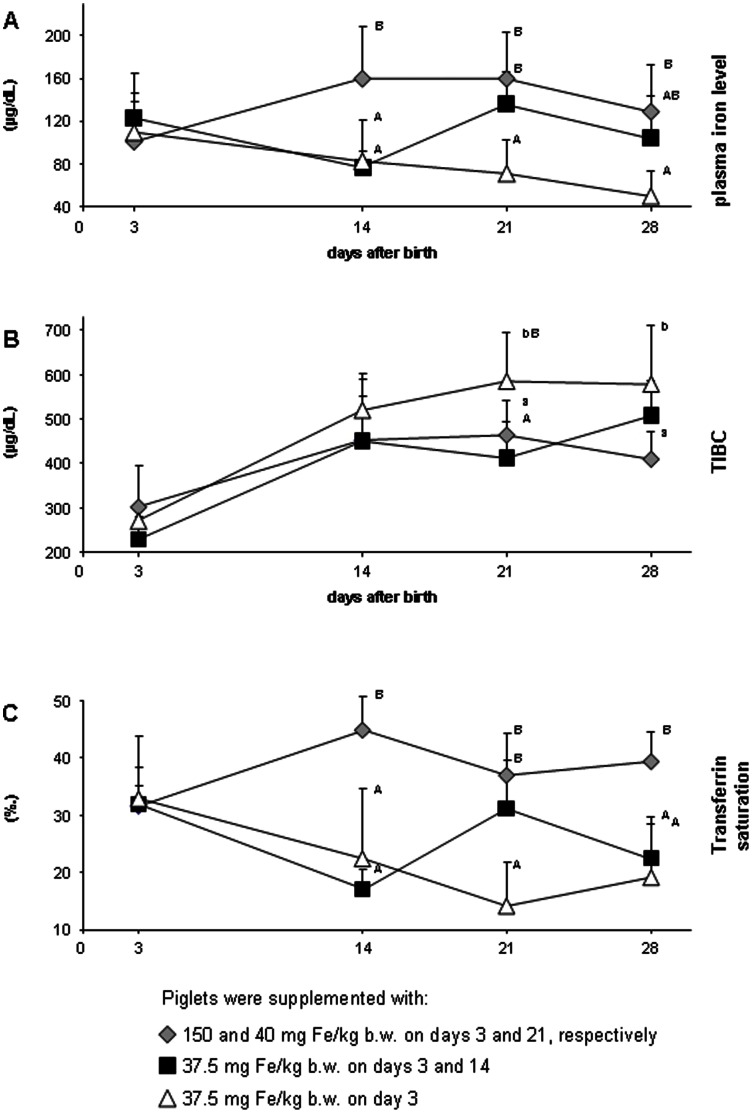
Plasma iron parameters of piglets supplemented with iron dextran according to various protocols. A; plasma iron level. B; total iron binding capacity. C; iron transferrin saturation. Values are expressed as the means ± S.D. Plasma iron parameters were determined for 9 piglets from each experimental group. Different capital and small letters denote statistically significant difference at P≤0.01 and P≤0.05, respectively, between parameters of piglets from different groups on a given day of experiment.

To assess the effect of different iron supplementation strategies on the iron status and hepcidin levels in piglets, three different protocols were employed. The control protocol involved two injections (split supplementation) of piglets with 150 and 40 mg Fe/kg b.w. on day 3 and 21 postpartum, respectively (group A), is a routine iron therapy of piglets applied at the farm where the study was performed. Our modified supplementation based on the protocol recently described [10] involved two injections of 37.5 mg Fe/kg b.w. on day 3 and 14 postpartum (group B). Piglets from the third group (group C) received 37.5 mg Fe/kg b.w. on day 3 only. As shown in Table 1, iron supplementation in group C was largely insufficient to maintain hematological parameters and plasma iron status (Figure 3) till the weaning (day 28). However, the rationale to include the latter group in this study was to determine how long this single injection could maintain the appropriate hematological status of piglets. Our results clearly show that after day 21, the values of all hematological parameters (except of RBC count) of piglets from group C were significantly lower compared with those form group A and B animals. Similarly, iron plasma parameters (Fe plasma level, TIBC and Tf saturation) indicated gradually developing iron deficiency in group C piglets. In contrast, RBC indices in animals from group A and B were in the range of standard piglet parameters in this age [22], [23]. Importantly, there were no differences in most hematological indices between piglets from group A and B throughout the experimental period. Significantly lower values in piglets from group B were recorded only for MCV on day 21 (P≤0.01), HCT on day 28 (P≤0.01) and MCHC on day 21 (P≤0.05) (Table 1). Similar hematological status of group A and B piglets strongly indicates that by applying a modified protocol with decreased amounts of supplemental iron, a satisfactory prophylactic effect preventing development of IDA in piglets can be achieved till weaning. This observation extends our previous results showing the efficacy of split supplementation with reduced amounts of iron till day 14 after birth [10].

**Table 1:** Hematological parameters (mean ± SD) of piglets supplemented with iron dextran according to various protocols.

Parameter	RBC (× 10^6^/µL)				HGB (g/dL)				HCT (%)				MCV(fL)				MCH (pg)				MCHC(g/dL)			
Age→ (days)Group ↓	3	14	21	28	3	14	21	28	3	14	21	28	3	14	21	28	3	14	21	28	3	14	21	28
**A**	**3.86**±0.72	**4.45**±0.59	**5.19**±0.78	**5.93**±0.72	**7.47**±1.43	**9.28**±1.18	**10.41**±1.41^B^	**11.49**±1.59^B^	**26.22**±4.09	**32.08**±2.40	**35.10**±4.94^b^	**37.39**±5.07^bB^	**68.28**±3.39	**72.46**±6.25	**67.95**±5.15^bB^	**63.11**±4.14^bB^	**19,37**±1,80	**20,96**±1.72B	**20,16**±1.41B	**19,42**±1.37B	**28,33**±1.70	**28,94**±1.04	**29,70**±1.22b	**30,78**±1.11B
**B**	**3.90**±0.41	**4.44**±0.59	**5.26**±0.80	**5.82**±0.75	**7.66**±0.79	**8.15**±0.88	**9.94**±1. 31^B^	**10.64**±1.36^B^	**27.18**±2.40	**7.47**±1.43	**34.56**±4.49^b^	**35.84**±4.02^aAB^	**69.88**±2.70	**66.15**±6.01	**66.14**±5.86^AB^	**61.80**±4.60^bB^	**19,70**±0,86	**18,43**±1.62A	**18,97**±1.63B	**18,29**±1.23B	**28.16**±0.47	**27.88**±0.54	**28.71**±0.62ab	**29.62**±0.88B
**C**	**3.98**±0.91	**4.85**±0.76	**5.17**±0.59	**5.94**±0.79	**7.65**±1.55	**8.8**±1.82	**7.92**±2.13^A^	**7.95**±2.22^A^	**26.27**±5. 30	**30.12**±5. 34	**29.70**±6.63a	**28.41**±6.46^aA^	**66.37**±3. 34	**62.09**±5.06	**53.74**±5. 34^aA^	**47.91**±9.02^aA^	**19,30**±1.09	**17,41**±1.93A	**15,21**±2.11A	**13,39**±3.24A	**29.10**±0.56	**27.98**±1.62	**28.22**±1.36a	**27.70**±1.74A

RBC – red blood cells count, HGB - hemoglobin level, HCT - hematocrit, MCV - mean cell volume, MCH - mean corpuscular hemoglobin, MCHC - mean corpuscular hemoglobin concentration. A, B, and C – experimental groups (n = 9 each) described in the legend to Figure 1. Hematological parameters were determined for 9 piglets from each experimental group. Different capital and small letters denotes statistically significant difference at P≤0.01 and P≤0.05, respectively, between parameters of piglets from different groups on a given day of experiment.

It is known that in iron deficiency, a drop of hematological parameters is preceded by a decrease in plasma iron level, Tf iron saturation and the increase in TIBC values [2]. This scenario was perfectly reproduced in group C piglets: decrease in plasma iron status recorded on day 14 resulted in a severe deterioration of hematological status on day 21. In group B piglets, the plasma iron status on day 14 was also found to be at the borderline of iron deficiency (note that at that time piglets from both groups received the same iron treatment), however, the second injection of iron to group B piglets just at that day restored efficiently plasma iron level and in consequence increased the values of most RBC indices.

**Figure 4:**
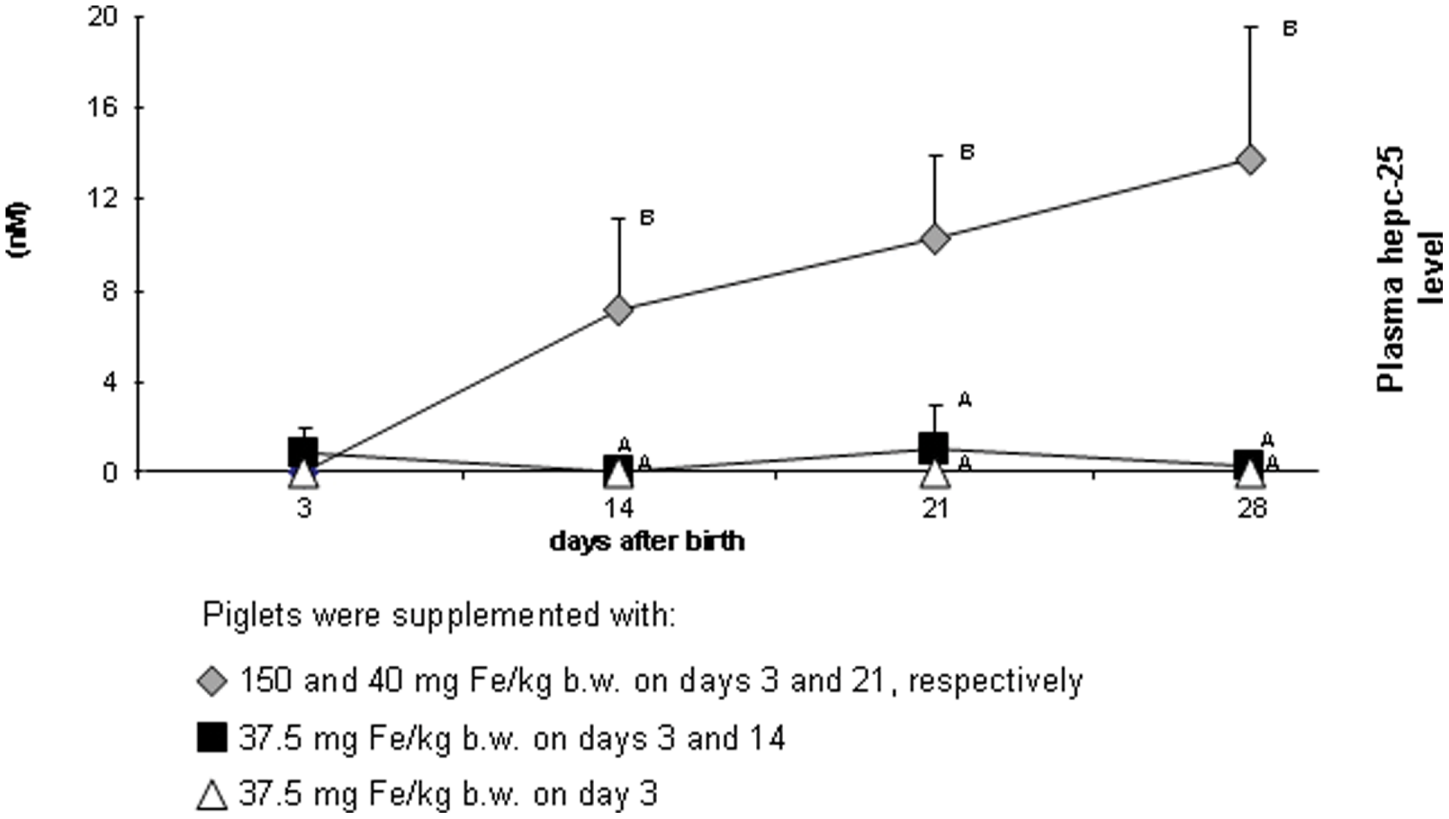
Hepcidin concentration in blood plasma of piglets supplemented with iron dextran according to various protocols. Values are expressed as the means ± S.D. Hepcidin concentration was determined for 5–7 piglets from each group/day. Different capital and small letters denotes statistically significant difference at P≤0.01 and P≤0.05, respectively, between parameters of piglets from different groups on a given day of experiment.

To assess the circulating levels of bioactive hepcidin in the circulation of the piglets, we developed and validated a novel mass spectrometry-based assay to quantifies hepcidin-25 in piglet plasma samples (see Material and Methods section for details). As shown in Figure 2, this methodology could detect the peptide peak that corresponds to pig hepcidin-25 of (with molecular weight of 2750 Dalton) in samples from group A piglets following injection of the high dose of iron, but not at baseline. In fact, the hepcidin-25 concentration in group A piglets showed in general a gradual increase throughout the experimental period (Figure 4). In contrast, the hepcidin-25 levels in plasma from group B and C piglets remained just above or below the lower limit of quantification (<1 nM) of our assay throughout the experimental period (Figures 4). The fact that we observe very low hepcidin plasma concentration measured in all 3 day-old piglets at baseline is surprising considering our previous finding of relatively high abundance of hepatic *hepcidin* mRNA levels in piglets of the same age, while no large increase in these liver mRNA levels were observed upon iron intervention [10]. This discrepancy may relate to an unknown hepcidin stimulus during early life in combination with immature hepatic hepcidin maturation and secretion pathways in these neonates [16] and shows the added value of actually assessing the active circulating peptide. On the other hand, the absence of circulating hepcidin-25 may relate to a relative low sensitivity of the mass spectrometry assay compared to mRNA quantification by real-time PCR in plasma from these young piglets, although this was not found for mouse bioactive hepcidin-25 [24]. Hepcidin concentration measured in plasma from group A piglets markedly increased after the first injection of high iron dose. Interestingly, in plasma of piglets supplemented with 37.5 mg Fe/kg b.w. on day 3 (group B and C), hepcidin-25 was hardly detectable up to day 14. Moreover, even after the second injection of iron dextran to piglets from group B on day 14, plasma hepcidin-25 levels continued to be very low till weaning (day 28). Notably, our methodology to quantify piglet hepcidin-25 yielded robust assay characteristics that were similar to those published for hepcidin-25 in human plasma samples [17]. For piglet hepcidin-25, intra-run coefficient of variations (CV) were 4.4% at 4.1 nM and 12.4% at 1.9 nM (n = 7), and inter-run CVs were 6.3% at 3.3 nM and 7.4% at 4.8 nM (n = 8). Dilution linearity of the piglet hepcidin-25 assay is shown in Figure S1 (R^2^ = 0.995).

Our results open an interesting field for speculations regarding the relationship between iron supplementation, regulation of iron metabolism and hematological status. Obviously, injection of high amounts of iron rectifies neonatal IDA in pigs as exemplified in this study and many others [10], [11], [12], [13]. However, it is accompanied by the risk of excessive hepcidin synthesis, which in turn may impair both the utilization of supplemental iron deposited in RES macrophages and the absorption of dietary iron. Our results clearly show that it is possible to strongly reduce the amount of supplemental iron, and still maintain values of RBC indices at the proper level without inducing hepcidin-25 expression. It is tempting to speculate that after injection of low doses of supplemental iron to piglets according to our modified protocol, iron is predominantly transferred to the bone marrow, where it ensures the correct course of erythropoiesis. In contrast, in piglets highly loaded with supplemental iron, iron is partially redistributed to hepatocytes, where it induces hepcidin synthesis. Hepatic iron is thought to induce hepcidin expression via bone morphogenetic protein (BMP) signaling [25], [26]. On the other hand when serum iron levels are low, transferrin receptor 1 (TfR1) sequesters HFE on hepatocytes, preventing its interaction with transferrin receptor 2 (TfR2), which is necessary for signaling to hepcidin. As serum iron saturation increases, HFE is displaced from its overlapping binding site on TfR1 by holo-Tf. HFE is then freed to interact with TfR2 and to signal for the increased production of hepcidin [27], [28]. Accordingly, after the first iron injection group A piglets maintain in parallel permanent high plasma iron levels, high Tf iron saturation and high hepcidin expression, which is then intensified by the second injection of iron dextran on day 21. An opposite relationship between iron plasma status and hepcidin levels occurs in iron deficient piglets from group C. Piglets from group B show an intermediate plasma iron status, which is sufficient to fuel erythropoiesis but does not reach a threshold indispensable for increasing plasma hepcidin levels.

Although our studies [10] demonstrate evident benefits of the modified protocol of split supplementation of newborn piglets with iron dextran, its usefulness in the swine industry may be limited because of the necessity for a second iron dextran injection, which makes this procedure labour-consuming and expensive. Our present results provide a molecular background for planning mixt parenteral/oral iron supplementation in newborn piglets. It seems that early (on day 3) bolus parenteral supplementation with reduced amount of iron dextran is indispensable because of the extreme iron deficiency in newborn piglets and the high iron demand for erythropoiesis. Keeping in mind that the injection to piglets of ∼40 mg Fe/kg b.w. does not induce hepcidin, we hypothesize that the second phase of iron supplementation with dietary iron starting on day 7–10 of life is an appropriate way to satisfy the iron requirements of piglets more physiologically. We have recently demonstrated that in piglets after day 4 postpartum, the two duodenal iron transporters – DMT1 and ferroportin are strongly expressed at their known site of activity in enterocytes [10]. Although, the effectiveness of exclusive dietary iron supplementation in improving hematological indices of piglets has been recently confirmed on a large population of animals [29], we still consider that the bolus administration of small amount of iron on day 3 is a necessary component of piglet supplementation. The concept of the innovative joined parenteral/oral supplementation of piglets with iron is now under investigation in our laboratory.

## Supporting Information

Figure S1 **Dilution linearity of the piglet hepcidin-25 assay.** A piglet plasma sample containing 8.3 nM (sample content 1.00) Hepcidin-25 was diluted 2, 3, 5 and 10 times in binding buffer (sample content 0.50, 0.33, 0.20 and 0.10, respectively) and applied to WCX-TOF MS. The measured hepcidin-25 values are indicated in nM. Dilution linearity: R^2^ = 0.995.
(TIF)

Table S1 (TIF)
