# Supplementary figures and images for: Iron Supplementation in Suckling Piglets: How to Correct Iron Deficiency Anemia without Affecting Plasma Hepcidin Levels
Source: PLoS One. 2013 May 30;8(5):e64022. doi: 10.1371/journal.pone.0064022 (PMC3667775; doi:10.1371/journal.pone.0064022)

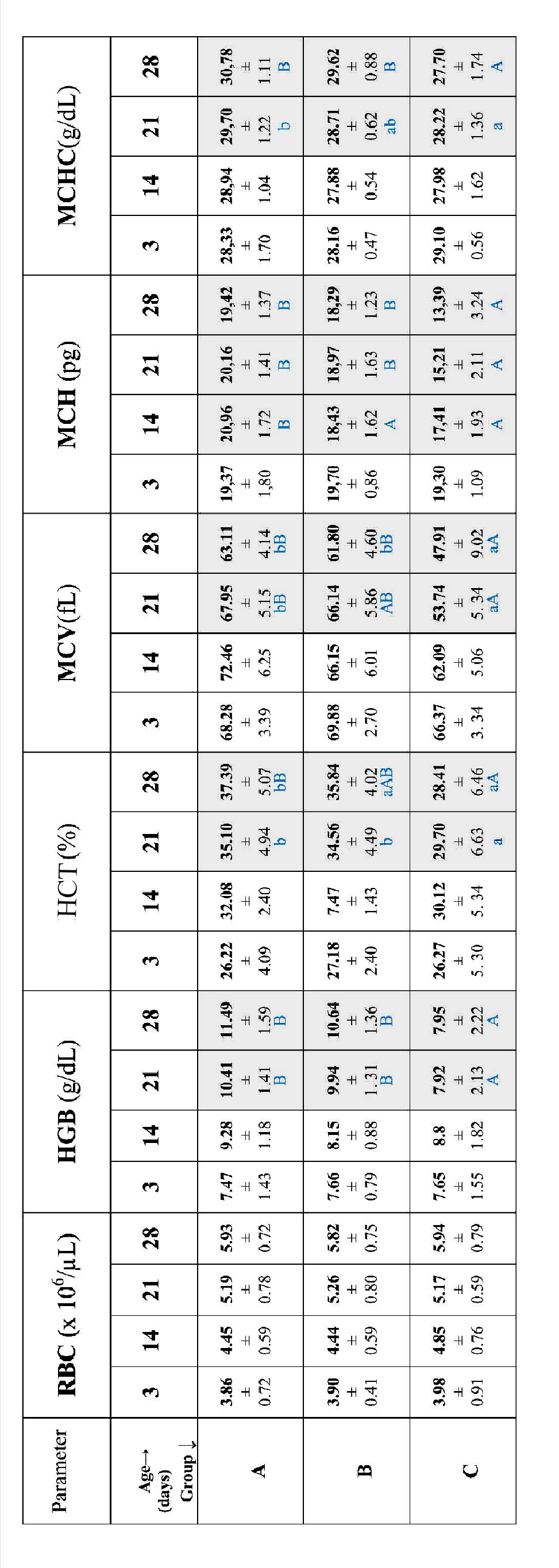

Supplement: Table S1 — (TIF) [file pone.0064022.s002.tif]
